# Rare Adverse Events Related to Nivolumab, an Immune Checkpoint Inhibitor: A Case Series

**DOI:** 10.7759/cureus.22070

**Published:** 2022-02-09

**Authors:** Nagapratap Ganta, Dina Alnabwani, Shawn Keating, Vraj Patel, Veera Jayasree Latha Bommu, Rand Dawoud, Pramil Cheriyath

**Affiliations:** 1 Internal Medicine, Hackensack Meridian Ocean Medical Center, Brick, USA; 2 Internal Medicine, Government Medical College and Hospital, Kadapa, IND; 3 Medical School, The Hashemite University, Amman, JOR

**Keywords:** checkpoint inhibitor related autoimmune diabetes mellitus (ciadm), adrenal insufficiency (ai), solid tumor, rectal carcinoma, melanoma, programmed death-1 (pd-1) inhibitors, immune checkpoint inhibitor, nivolumab related adverse events

## Abstract

Immune checkpoint inhibitors are a novel class of immunotherapy drugs that have improved the prognosis of melanoma, renal cell carcinoma, non-small cell lung cancer, urothelial carcinoma, and various other solid tumors. Nivolumab is an immune checkpoint inhibitor that acts by inhibiting programmed death. Its use is associated with significant immune-related adverse events, such as pneumonitis, thyroiditis, hepatitis, pruritus, vitiligo, and diarrhea. However, adrenal insufficiency and checkpoint inhibitor-related autoimmune diabetes mellitus are extremely rare adverse events related to nivolumab treatment. Here, we are highlighting cases of adrenal insufficiency and diabetes inspidus as a result of nivolumab. These rare adverse events in our case series are to raise awareness that this medication also may be the cause for this illness among oncologists, endocrinologists, internists, and other clinicians.

## Introduction

With recent advancements in immunology and cancer biology, new classes of immunomodulatory therapies have been developed to aid tumor management [1]. Immune checkpoint inhibitors (ICIs) have improved the prognosis of melanoma, renal cell carcinoma, non-small cell lung cancer, urothelial carcinoma, and other solid tumor types. Ipilimumab (cytotoxic T-lymphocyte-associated antigen 4: CTLA-4 inhibitor), nivolumab, and pembrolizumab (programmed death: PD-1 inhibitors), atezolizumab, avelumab, and durvalumab are some of the medications in this class (programmed death-ligand: PD-L1 inhibitors) that have been approved. 

Despite their key role in regulating the immune response by either stimulating or inhibiting the pathways that modulate T-cell function, ICIs are associated with serious immune-related adverse events (irAEs), which can affect any organ and are difficult to diagnose and treat [2]. Rashes, pruritus, vitiligo, thyroiditis, diarrhea, hepatitis, and pneumonitis are among the most common immune-related side effects of PD-1 inhibitors. Nivolumab is usually associated with pneumonitis and thyroid dysfunction and only rarely causes adrenal insufficiency (AI) or elevated blood sugar levels (0.7% of patients in randomized clinical trials) [3].

Here, we report a case series of nivolumab-induced AI and diabetes mellitus in a 79-year-old male patient and a 62-year-old female patient, respectively. 

## Case presentation

Case summary 1

A 79-year-old male presented to the emergency department with complaints of dizziness, myalgia, and nausea associated with a decreased appetite for 4 days prior to presentation. He denied any associated fever, vomiting, abdominal pain, trauma, diplopia, or numbness. He has a history of hypertension, benign prostatic hyperplasia, malignant melanoma of the buccal mucosa, and rectal cancer. He was currently receiving immunotherapy consisting of nivolumab (Opdivo®, Bristol-Myers Squibb Company, New York, USA) every 4 weeks for rectal cancer. He developed a progressive generalized rash over the month prior to presentation for which he was evaluated by a dermatologist who diagnosed him with atopic dermatitis. 

The patient’s vital signs on presentation were as follows: temperature, 98.1°F; pulse rate, 83/min; blood pressure, 128/71 mmHg; and respiratory rate, 18 breaths per minute with 99% SpO2 on room air. On physical examination, the patient was alert and oriented to place, person, and time, and slight edema in the lower extremities was observed. His laboratory results were as follows: sodium, 122 mmol/L; potassium, 4.4 mmol/L; chloride, 91 mmol/L; blood glucose, 88 mg/dL; and blood urea nitrogen, 8 mg/dL. The calculated serum osmolarity for the patient was 251.75 mOsm/L. His baseline serum sodium typically ranges between 138 and 140 mEq/L on his monthly labs. His creatinine level was 0.83 mg/dL. He was diagnosed with symptomatic hypoosmolar hyponatremia. The patient was started on 1000 mL of normal saline, 2.5 mg oral amlodipine daily, and 40 mg enoxaparin subcutaneously every 24 h. A nephrologist and oncologist were consulted. His serum cortisol level was 0.8 mcg/dL (range 8.7- 22.4 mcg/dL) and his adrenocorticotropic hormone (ACTH) level was 9 pg/mL (range 6-50 pg/mL). Based on laboratory values, symptoms and after ruling out other possible causes for AI, it was suspected that it is a medication-induced AI, most likely due to nivolumab. Intravenous hydrocortisone at a dose of 50 mg was administered every 8 h. He was discharged once his sodium level was normalized at which point daily hydrocortisone at a dose of 20 mg in the morning and 10 mg in the afternoon was initiated. The patient was followed up by a nephrologist, endocrinologist, and oncologist.

Case summary 2

The patient was a 62-year-old female with a history of metastatic melanoma to the lymph node (stage IIIa) and right breast cancer of the upper quadrant; she underwent a lumpectomy, received radiotherapy, and presented to our office with the concern of newly elevated blood sugar. 

She was previously diagnosed with right breast cancer of the upper quadrant in 2008 and was treated with tamoxifen for 5 years. She has since remained in complete remission. This patient was then diagnosed with malignant melanoma in situ on biopsy from the left lower extremity which spread to the lymph node (stage IIIa) in 2019 and received nivolumab for 1 year. She also has a history of obesity, anxiety, asthma, leukopenia, mixed hyperlipidemia, and osteoarthritis. She has no known allergies, and her family history was significant for breast cancer, which affected her sister. The patient was a former smoker for 8 years and denied the current use of smokeless tobacco and recreational drugs. However, she reported current alcohol use. Besides 480 mg of nivolumab every 4 weeks since 2019, her current medications included triamcinolone 0.5% cream, alprazolam 0.5 mg, rosuvastatin 5 mg, albuterol HFA (hydrofluoroalkane) as needed for wheezing, budesonide-formoterol fumarate 80-4.5 mcg/ACT inhaler, lisinopril 5 mg tablet, and cholecalciferol.

She underwent positron emission tomography-computed tomography (PET/CT) scanning of the whole body, which did not reveal any evidence of recurrent or metastatic disease, and no significant change to her previous PET/CT in 2020 was observed. Her blood work revealed a white blood cell count of 4,500 cells/mm3, an absolute neutrophil count of 2.6 cells/µL, hemoglobin of 12.5 g/dL, hematocrit of 38.2%, a platelet count of 191,000 cells per mm3 of blood, random glucose level of 563 mg/dL, hemoglobin A1C of 10.2%, C-peptide of 0.11 (range 0.8-5.2 ng/mL), microalbumin <0.5 mg/dL, and a urinary creatinine level of 33.39 mg/dL; her lipid profile was within normal range.

She was diagnosed with drug (nivolumab)-induced diabetes mellitus and started on insulin therapy. She initiated insulin glargine (Lantus®, sanofi‐aventis US LLC, Bridgewater Township, USA) at 25 units twice daily and insulin glargine (Basaglar® KwikPen®, Lilly, Indianapolis, USA) at 20 units in the morning and 15 units in the evening. After a month of follow-up, her glucose readings were under control.

## Discussion

Traditional cytotoxic chemotherapy is being replaced by tailored cytostatic medicines that target certain cancer-related biological targets. Checkpoint ligands, which are cell surface molecules found on some normal cells and many tumor cells, interact with checkpoint receptors, such as CTLA-4 and PD-1, on T cells. T-cell activation is negatively regulated by activated checkpoint receptors, which are important in not only limiting inflammation and autoimmunity but also in tumor evasion from the host [1].

Nivolumab, a monoclonal antibody against PD-1, is currently used to treat various advanced malignancies, including recurrent and metastatic head and neck cancer. However, increasing numbers of studies have reported immune-related side effects after nivolumab therapy [4]. Nivolumab is a completely humanized immunoglobulin (Ig) G4 monoclonal antibody that targets the PD-1-negative immunoregulatory cell surface receptor and that possesses immunosuppressive and anticancer properties. Nivolumab binds to and inhibits the activation of PD-1, a transmembrane protein belonging to the Ig superfamily that is overexpressed in cancer and antigen-presenting cells. Nivolumab inhibits PD-1, which results in T-cell activation and cell-mediated immune responses against tumor cells. Increased autoimmunity, including autoimmune endocrine dysfunction, is an expected side effect because the method of action includes boosting T-cell responses [1].

The inability of the adrenal gland cortex to produce sufficient levels of glucocorticoids and/or mineralocorticoids is known as primary adrenal insufficiency (PAI). This scenario is potentially life-threatening due to the vital role that glucocorticoids and mineralocorticoids play in salt, fluid, and energy balance. The hypothalamic-pituitary axis responds to lower serum cortisol levels by increasing ACTH production [2]. Some of the causes of PAI are autoimmune, infectious, genetic, metastasis, drug-induced. The common drugs causing PAI are warfarin, phenobarbital, phenytoin, rifampin, ketoconazole, fluconazole [5]. PAI is considered extremely rare among irAEs, but it may be underdiagnosed. 

A systematic review and meta-analysis published in 2018 included 38 randomized trials with 7,551 patients and reported the incidence of endocrinopathies caused by ICIs. According to that study, PAI of any grade was documented in 43 out of 5,831 individuals (0.7%), although only 0.2% had grade 3 or above. AI was more common in patients who received a combination of ICIs (11 of 262, 4.2%) [6]. Wang et al. [7] analyzed fatal toxicity data from a global adverse drug reaction database (VigiLyze, VigiBase) and found 614 fatal irAEs; adrenal toxicity was the cause of death in eight patients treated with ipilimumab and six patients treated with anti-PD-1/PD-L1 drugs [8].

AI can manifest in various ways, ranging from asymptomatic test changes to a significant medical emergency. Fatigue, dizziness, orthostatic symptoms, anorexia, weight loss, and stomach discomfort are the most common clinical symptoms, just as they are with AI due to any other cause. Refractory hypotension, altered state of consciousness, widespread weakness, stomach discomfort, shock, and, in severe cases, death are common symptoms. Hyponatremia and hyperkalemia are common laboratory abnormalities, but hypoglycemia and hypercalcemia are less common [2]. Early morning cortisol, ACTH, and ACTH stimulation tests are all laboratory tests to consider, as they could show the degree of AI. Additional tests, such as those that determine renin and aldosterone levels after ACTH stimulation, can help evaluate the severity of the mineralocorticoid deficit [2].

PD-L1 and PD-L2 were recently discovered to be expressed in non-immune tissues in mice, including the anterior and intermediate portion of the pituitary gland, extending to the ventral side of the pituitary stalk, but not the posterior pituitary gland [9]. This shows that a specific lesion could occur that exclusively affects certain anterior pituitary cells, such as those that produce ACTH. To understand the pathophysiology of pituitary-specific toxicity associated with PD-1 inhibitors, it is crucial to understand how PD-1 is expressed in the pituitary [1].

Nivolumab-induced autoimmune diabetes mellitus is another rare adverse event. Type I diabetes mellitus (T1DM) is diagnosed by the occurrence of hyperglycemia with evidence of autoimmunity and insulin insufficiency; 90% of patients test positive for islet autoantibodies at some point during treatment [10]. Patients with drug reactions with eosinophilia and systemic symptoms (DRESS) are at risk for autoimmune disorders such as T1DM, thyroiditis, and systemic lupus erythematosus (SLE). Other drugs causing T1DM are carbamazepine, mexiletine, minocycline, allopurinol, and dapsone [11].

The best predictor of the development of checkpoint inhibitor-related autoimmune diabetes (CIADM) is exposure to ICI therapy involving the PD-1/PD-L1 axis. According to pharmacovigilance data from the FDA Adverse Events Reporting System, adverse events are highest after the administration of anti-CTLA-4 plus either anti-PD-1 or anti-PD-L1 therapy (2.60%), followed by anti-PD-1 therapy alone (1.18%), anti-PD-L1 therapy alone (0.73%), and anti-CTLA-4 therapy alone (0.33%) [12].

Recent research has revealed that the autoimmune destruction of the pancreas is caused by a flaw in the beta-cell insulin gene sequence. This event, which is most noticeable in cancer patients who receive immunotherapy treatment, demonstrates that previously working beta cells begin to express non-functional proteins, a characteristic trait of cancer cells. This triggers the usual antitumor reaction of the immune system against these pancreatic cells, which could explain why some cancer patients develop T1DM after receiving immunotherapy [13]. Anti-PD-1-related T1DM cases have yielded mixed results in terms of the prevalence of diabetes-related autoantibodies after the onset of T1DM. Hughes et al. reported that, after receiving anti-PD1 medication, five cases exhibited new-onset insulin-dependent diabetes. After the onset of T1DM, three of these five patients were positive for at least one diabetes-related autoantibody [14].

The number of patients who will receive anti-PD1 therapy will rapidly increase as its approval expands in the upcoming years, as will the frequency of the autoimmune side effects. Medical oncologists should be aware of the risk of anti-PD1 therapy-induced autoimmune disease and advise their patients to report any symptoms as soon as possible, as this constitutes a medical emergency. Treatment of CIADM is similar to treatment of T1DM and includes insulin regimens according to the patient’s serum glucose levels. Patients should be treated early to prevent life-threatening complications such as diabetic ketoacidosis (DKA).

Today, recognition of irAEs in cancer patients is critical, as individuals may present without conventional symptoms or, as in our case, with isolated endocrine abnormalities. Since irAEs are classified as hazardous adverse events, most recommend stopping immune checkpoint inhibitor treatment, but this remains debatable. In most individuals with AI, high-dose glucocorticoid treatment is also recommended, while an insulin regimen is recommended for T1DM [1].

## Conclusions

Advances in immuno-oncology have resulted in the development of successful treatments consisting of novel immune response-modulating drugs. irAEs are common when ICIs are frequently used. If not detected and treated early, AI or CIADM can have very catastrophic consequences. Therefore, it is critical to pay close attention to these possibly fatal unusual consequences and maintain a high level of clinical suspicion. Lifelong glucocorticoid treatment is the treatment of choice for PAI to restore the hypothalamic-pituitary-adrenal axis, while insulin regimens are recommended for CIADM. Long-term monitoring is required as is patient education so that symptoms are identified; proper counseling is also necessary to control and increase awareness of potential side effects.
